# The 1,4 benzoquinone-featured 5-lipoxygenase inhibitor RF-Id induces apoptotic death through downregulation of IAPs in human glioblastoma cells

**DOI:** 10.1186/s13046-016-0440-x

**Published:** 2016-10-22

**Authors:** S. Zappavigna, M. Scuotto, A. M. Cossu, D. Ingrosso, M. De Rosa, C. Schiraldi, R. Filosa, M. Caraglia

**Affiliations:** 1 Department of Biochemistry, Biophysics and General Pathology, Second University of Naples, via L. De Crecchio 7, Naples, 80138 Italy; 2 Department of Experimental Medicine, Second University of Naples, Via L. De Crecchio, 7, Naples, 80138 Italy

**Keywords:** Apoptosis, Glioblastoma, IAPs, NFκB, **RF-Id**

## Abstract

**Background:**

Embelin is a potent dual inhibitor of 5-lipoxigenase (5-LOX) and microsomal prostaglandin E2 synthase (mPGES)-1 that suppresses proliferation of human glioma cells and induces apoptosis by inhibiting XIAP and NF-κB signaling pathway. Synthetic structural modification yielded the derivative 3-((decahydronaphthalen-6-yl)methyl)-2,5-dihydroxycyclohexa-2,5-diene-1,4-dione (**RF-Id**), an embelin constrained analogue, with improved efficiency against 5-LOX in human neutrophils and anti-inflammatory activity in vivo. Taking into account that lipoxygenase (LOX) metabolites, from arachidonic acid and linoleic acid, have been implicated in tumor progression, here, we determined whether **RF-Id** was able to hinder glioblastoma (GBM) cancer cell growth and the related mechanisms.

**Methods:**

U87MG and LN229 cells were plated in 96-wells and treated with increasing concentrations of **RF-Id**. Cell viability was evaluated by MTT assay. The effects of the compounds on cell cycle, apoptosis, oxidative stress and autophagy were assessed by flow cytometry (FACS). The mode of action was confirmed by Taqman apoptosis array and evaluating caspase cascade and NFκB pathway by western blotting technique.

**Results:**

Here, we found that **RF-Id** induced a stronger inhibition of GBM cell growth than treatment with embelin. Flow cytometry analysis showed that **RF-Id** induced about 30 % apoptosis and a slight increase of autophagy after 72 h on U87-MG cells. Moreover, the compound induced an increase in the percentage of cells in G2 and S phase that was paralleled by an increase of p21 and p27 expression but no significant changes of the mitochondrial membrane potential; array analysis showed a significant upregulation of *CASP8* and a downregulation of *IAP* family and *NFκB* genes in cells treated with **RF-Id. RF-Id** induced a significant cleavage of caspases 8, 9, 3 and 7, blocked c-IAP2/XIAP interaction by inducing XIAP degradation and inhibited NFκB pathway.

**Conclusions:**

**RF-Id** induced a caspase-dependent apoptosis in GBM cells by inhibiting IAP family proteins and NFκB pathway and represents a promising lead compound for designing a new class of anti-cancer drugs with multiple targets.

**Electronic supplementary material:**

The online version of this article (doi:10.1186/s13046-016-0440-x) contains supplementary material, which is available to authorized users.

## Background

Glioblastoma multiforme (GBM) is the most common and deadliest of malignant primary brain tumors in adults. Combination of radiotherapy and temozolomide (TMZ) represents the gold-standard first-line treatment for GBM [1, 2]. In recent phase II trials, also nitrosoureas such as fotemustine (FTM) have shown mild activity for recurrent GBM [3, 4]. The blood–brain barrier (BBB) is the most important limiting factor for the development of new drugs and drug delivery for the central nervous system (CNS) [5]. In this context, we showed that the newly developed transferrin (Tf)-targeted self-assembling nanoparticles (NPs) incorporating zoledronic acid (ZOL) allowed the successfully use of ZOL in the treatment of GBM, potentiating its in vitro and in vivo antitumor activity on GBM through the acquisition of ability to cross the BBB [6].

Despite the aggressive first-line therapy and the recent results with surgery, radiation therapy and adjuvant chemotherapy, tumors invariably recur and median survival is 15 months [7]. Further progress towards a cure for malignant gliomas will require a greater understanding of the underlying mechanisms driving the growth and resistance to therapy of these tumors.

In this framework, it is well established that many cancers derive from sites of chronic inflammation; the tumor environment, which is orchestrated by the interplay between inflammatory cells, tumor cells, and other tumor-associated host cells, is an essential member in the tumorigenic progression. Cancer and inflammation are intimately linked due to specific oxidative processes in the tumor microenvironment.

In this context, the pro-inflammatory microenviroment in various human brain tumors is characterized by high expression of 5-Lipoxygenases (5-LOX) [8], a versatile class of oxidative enzymes involved in arachidonic acid metabolism, that promote the proliferation of glioma cells [9–12]. In addition, is well recognized that the inhibitors of 5-LOX can activate Caspase-3 inducing glioma cell apoptosis, suggesting that 5-LOX can participate in the survival of glioma cells [13]. In this context NDGA, a natural 5-LOX inhibitor, and its methylated derivative, terameprocol, exhibited anticancer activity; moreover, terameprocol is a global transcription inhibitor that affects cell division, apoptosis, drug resistance, hypoxia responsive genes, and radiation resistance in hypoxia. Previous research indicated terameprocol as a drug that could be safely combined with radiation in newly diagnosed high-grade glioma [14].

Starting from these considerations, as cancer is a multifactor disease, there is great interest in the development of new compounds able to target multiple intracellular components.

Our group have been interested for a long time in the synthesis and the biological evaluation of anti-cancer and anti-inflammatory agents including quinone-based compounds [15–26]. Within the context of our investigations towards the synthesis of quinone derivatives with prospects for therapeutic use, we recently studied the natural compound embelin, a potent dual inhibitor [18] of 5-lipoxigenase (5-LOX) and microsomal prostaglandin E2 synthase (mPGES)-1 able to suppress proliferation of human glioma cells and to induce apoptosis by inhibiting XIAP and NF-κB signaling pathway [27–29].

Moreover, we have previously discovered (**RF-Id**), an embelin constrained analogue, with improved efficiency against 5-LOX in human neutrophils and anti-inflammatory activity in vivo [15, 19], and the aim of the present study was to determine the precise cellular mechanisms of the new 5-LOX inhibitor **RF-Id** and verify its intrinsic inhibition of XIAP function.

## Methods

### Materials

All reagents were analytical grade and purchased from Sigma Aldrich (Milano, Italy). RPMI, DMEM were purchased from Life Technologies (Carlsbad, CA) supplemented with 10 % FBS (fetal bovine serum), 1 % penicillin, streptomycin and L-glutamine from Lonza Group Ltd (Svizzera). The rabbit antibodies raised against α − tubulin, caspase-3, detecting endogenous levels of full length caspase-3 (35 kDa) and the large fragment of caspase-3 resulting from cleavage (17 kDa); caspase-8, detecting endogenous level of total caspase-8 (57 kDa), including the p10 subnit of the activated protein; caspase-9, detecting endogenous levels of full length caspase-9 (47 kDa), a large fragment (35 kDa or 17 kDa) and a small fragment (10 kDa) of caspase-9 resulting from cleavage; p21, p27, XIAP, C-IAP-2, pIKKα/β, IKKα/β, pIKBα, IKBα and p65 subunit of NFκB were purchased by Cell Signaling Technology. The rabbit antibody raised against caspase -7 detecting endogenous levels of full length caspase-7 (34 kDa) was purchased by Santa Cruz Biotechnology.

### Chemistry

The compound **RF-Id** was synthesized and characterized as reported previously [18]. **RF-1d-met** was prepared starting from 1,2,4,5-tetramethoxybenzene and decahydronaphthalene-2-carbaldehyde. Coupling the above aldehyde with the lithium anion of 1,2,4,5-tetramethoxybenzene (2.5 equiv of BuLi, THF) afforded compound **RF-1d-met** as a mixture of diastereomers in 65 % yield.

### Cell culture

Human GBM cell lines U87MG and LN229 were provided by Dr. Carlo Leonetti (Regina Elena Cancer Institute, Rome, Italy). U87MG and LN229 cells were grown in RPMI and DMEM, respectively, supplemented with 10 % heat-inactivated fetal bovine serum, 20 mM HEPES, 100 U/mL penicillin, 100 mg/mL streptomycin, 1 % L-glutamine and 1 % sodium pyruvate. Cells were cultured at 37 °C in a 5 % CO_2_ −95 % air environment in a humidified incubator.

### Cell proliferation assay

Cells were seeded in serum-containing media in 96-well plates at the density of 2 × 10^3^ cells/well. After 24 h incubation at 37 °C, cells were treated with increasing concentrations of **RF-Id**, embelin and reference compounds (0,8–100 μM) for 24 h, 48 h and 72 h. Cell viability was assessed by adding MTT [3-(4,5-dimethylthiazol-2-yl)-2,5-diphenyl tetrazolium bromide] solution in phosphate-buffered saline (PBS) to a final concentration of 5 mg/mL. The plates were then incubated at 37 °C for an additional 4 h and the MTT-formazan crystals were solubilized in 1 N isopropanol/hydrochloric acid 10 % solution at 37 °C on a shaking table for 20 min. The absorbance values of the solution in each well were measured at 570 nm using a Bio-Rad 550 microplate reader (Bio-Rad Laboratories, Milan, Italy). Percentage of cell viability was calculated as the following formula (absorbance of the treated wells - absorbance of the blank control wells)/(absorbance of the negative control wells - absorbance of the blank control wells). All MTT experiments were performed in quadruplicate.

### Flow cytometric analysis of cell cycle

U87MG cells were seeded in 6-well plates in a number of 2 × 10^5^ cells per well and were treated 24 h later with the concentration inhibiting 50 % of cell growth (IC:50) of **RF- Id**. After 72 h of treatment, cells were washed in PBS and directly stained in a PI solution (50 μg PI in 0.1 % sodium citrate, 0.1 % NP40, pH 7.4) for 30 min at 4 °C in the dark. Flow cytometry analysis was performed using a BD Accuri™ C6 (Becton Dickinson). To evaluate cell cycle, PI fluorescence was collected as FL3 (linear scale) by the ModFIT software (Becton Dickinson). For the evaluation of intracellular DNA content, at least 20,000 events for each point were analyzed in at least three different experiments giving a SD less than 5 %.

### Flow cytometric analysis of apoptosis

Apoptotic cell death was analysed by Annexin-V–FITC staining and by propidium iodide (PI) detection systems (eBioscences, Vienna, Austria). Briefly, U87MG cells were seeded in 6-well plates in a number of 2 × 10^5^ cells per well and were treated 24 h later with Rf-Id IC:50. After 72 h of treatment cells were trypsinezed, washed twice with PBS 1X and pellets were resuspended in 200 μL Binding Buffer 1X. Then, 5 μL Annexin V-FITC were added to 195 μL cell suspension, mixed and incubated for 10 min at room temperature. Cells were washed with 200 μL Binding Buffer 1×, resuspended in 190 μL Binding buffer 1× and 10 μL Propidium Iodide (20 μg/mL) was added. The detection of viable cells, early apoptosis cells, late apoptosis cells and necrotic cells were performed by BD Accuri™ C6 (Becton Dickinson). For each sample, 2 × 10^4^ events were acquired. Analysis was carried out by triplicate determination on at least three separate experiments.

### Western blot analysis

U87MG cell lines were treated with **RF-Id** (IC:50) for 24 h, 48 h and 72 h or Bortezomib (500nM) for 24 h or a combination of Bortezomib and **RF-Id** for 24 h and 72 h**,** respectively at 37 °C. For cell extract preparation, cells were washed twice with ice-cold PBS, trypsinized and centrifuged for 30 min at 4 °C in 1 ml of lysis buffer (1 % Triton, 0.5 % sodium deoxycholate, 0.1 NaCl, 1 mM EDTA, pH 7.5, 10 mM Na_2_HPO_4_, pH 7.4, 10 mM PMSF, 25 mM benzamidin, 1 mM leupeptin, 0.025 units/ml aprotinin). Equal amounts of cell proteins were separated by SDS-PAGE in a sample buffer 1× (Sample Buffer 5X: Tris 10 mg/ml, SDS 30 mg/ml, b-mercaptoethanol 0.15 ml, glycerol 0.3 ml, bromophenol blue), electrotransferred to nitrocellulose membrane. Membrane were washed in TBST (10 mM Tris, pH 8.0, 150 mM NaCl, 0,05 % Tween 20), and blocked with TBST supplemented with 5 % nonfat dry milk. Then, membranes were incubate with primary antibodies in TBST and 5 % nonfat dry milk, washed, and incubated with horseradish peroxidase-conjugated secondary antibodies. All primary Abs were used at a diluition of 1:1000, instead all secondary Abs were used at a diluition of 1:2000. Blots were then developed using enhanced chemiluminescence detection reagents ECL (Thermo Fisher Scientific, Rockford, IL) and exposed to x-ray film. All films were scanned by using Quantity One software (BioRad Chemi Doc).

### Flow cytometric analysis of mitochondrial potential (Mitotracker Red)

Changes in mitochondrial membrane potential due to ROS generation was analyzed by flow cytometry by using Mitotracker Red CMXRos (INVITROGEN Carlsbad, California, Stati Uniti). Briefly, U87MG and LN229 cells were plated in 6-multiwell plates at the density of 2 × 10^5^ cells/well. After treatment, cells were detached, centrifuged at 1200 g for 5 min and resuspended in PBS. Mitotracker Red probe was added at a final concentration of 100 nM. The latter does not emit fluorescence if it is not internalized in living cells. Mitotracker Red is oxidised in presence of ROS and emits a red fluorescence (FL2 channel) directly proportional to the potential of mitochondrial membrane. After the incubation with probe for 20 min at room temperature in the dark, cells were centrifuged, resuspended and analysed by BD Accuri™ C6. For each sample, 20,000 events were acquired. Analysis was carried out by triplicate determination on at least three separate experiments.

### Flow cytometric analysis of autophagy

Autofagy was analyzed by flow cytometry by using monodansylcadaverine (MDC)(Sigma-Aldrich, Saint Louis, Missouri, Stati Uniti) staining. MDC is an auto-florescent agent used as a selective marker for autophagic vacuoles (AVOs) and especially autolysosomes. U87MG cells were plated in 6-multiwell plates at the density of 2 ×10^5^ cells/well. After 72 h of treatment cells were incubated with 50 μM of MDC in PBS 1x at 37 °C for 15 min. After the incubation the cells were washed twice in PBS 1×, trypsinized and resuspended in 500 μl of PBS 1×. Analysis was performed by flow cytometric (BD Accuri™ C6). The fluorescent emissions were collected through FL1 channel. For the quantitative evaluation of MDC, BD Accuri™ C6 software (Becton Dickinson) was used to calculate MFIs. The MFIs were calculated by the formula (MFI treated/MFI control), where MFI treated is the fluorescence intensity of cells treated with the various compounds and MFI control is the fluorescence intensity of untreated and unstained cells. For each sample, 20,000 events were acquired. Analysis was carried out by triplicate determination on at least three separate experiments.

### RNA extraction and Taqman Human Apoptosis Array-Real-Time-PCR

Total RNA was prepared from U87MG cells according to mirVana PARIS (Ambion) protocol. In details, cells were centrifuged at 1,500 rpm for 5 min; the pellets obtained were placed on ice and then resuspended in 300 μl of Disruption Buffer. Successively, an equal volume of Denaturing Buffer (previously set at 37 °C) was added to the pellets and they were incubated in ice for 5–10 min. Then, Phenol-chloroform was added in the tube in a volume equal to the total volume and centrifugated at 10,000 rcf for 5 min. After centrifugation, the aqueous phase was collected and a volume of absolute ethanol equal to 1.25 times that of the collected volume was added. Then 700 μL at a time were collected, applied in Filter Cartridge and centrifuged at 10,000 rcf for 5 min. 700 μL of Wash Solution 1 were added on each Filter Cartridge, followed by centrifugation at 10,000 rcf for 40 s, later two washings with 500 μl of Wash Solution 2 were performed. Finally, 50 μl of Eluition Buffer (preheated at 95 °C) were added to the Filter Cartridge, previously placed on new tubes. Then, the samples were quantized to Nanodrop (Technologies Inc., Wilmington, DE). The cDNA was obtained by using QuantiTect Reverse Transcription Kit, (Quiagen) according to the manufacturer’s instructions. PCR products for all the genes tested were assessed in triplicate wells using TaqMan pre-developed assay reagents. The arithmetic standard normalization procedures recommended by the Data Assist software for microarray data were followed. In brief, data transformation was corrected for the signal from the three endogenous controls (18S, ACTB, GAPDH). Per card (mean) normalization accounted for the variability of each card by dividing all of the measurements on each card by the value of the 50th percentile. Per gene normalization accounted for the variability between probe sets for the three reporter genes. The threshold cycle, Ct, was automatically assigned by the SDS2.2 software package (Applied Biosystems). Relative quantities (RQ) were determined using the equation RQ = 2 − ΔΔCt. All data were generated in triplicate (different TLDA plates) and expressed as the mean ± SD. Differentially expressed genes were selected from the normalized data using a procedure known as significance analysis of microarrays, as installed in the SDS2.3 software package (Applied Biosystems). Genes were considered to be significant when their average fold change (FC) was ≤ −1.5 or ≥1.5, and statistically significant when the corresponding *p* value was ≤0.05. Two software programs were used to analyze the data, namely SDS RQ Manager 1.2 and DataAssist v.2.2 software (Applied Biosystems).

Taqman human apoptosis array contains 93 human genes in addition to 3 endogenous controls (18S, ACTB, GAPDH). Real-time quantitative PCR was performed on a ViiA7™ Real time PCR system (Applied Biosystems, Darmstadt, Germany). Relative expression of the transcripts was measured by using ViiA7™Real-Time PCR software (Applied Biosystems, Darmstadt, Germany). Treated samples were normalized to the corresponding medium-only control.

### Immunoprecipitation

Total protein extracts were subjected to immunoprecipitation with 2 μg of anti-XIAP or anti-cIAP2 for 24 h at 4 °C. Immune complexes were collected with 50 μl of protein A-agarose for 16 h at 4 °C. The protein A-agarose/immune complex was washed twice with cold PBS, resuspended in 20 μl of SDS-loading buffer, heated to 95 °C for 5 min and used for Western blotting analysis using anti-XIAP or anti-CIAP2.

### Statistical analysis

All data are expressed as mean + SD. Statistical analysis was performed by analysis of variance (ANOVA) with Neumann-Keul’s multiple comparison test or Kolmogorov-Smirnov test where appropriate.

## Results

### Effects of RF-Id on the proliferation of GBM cells

**Fig. 1:**
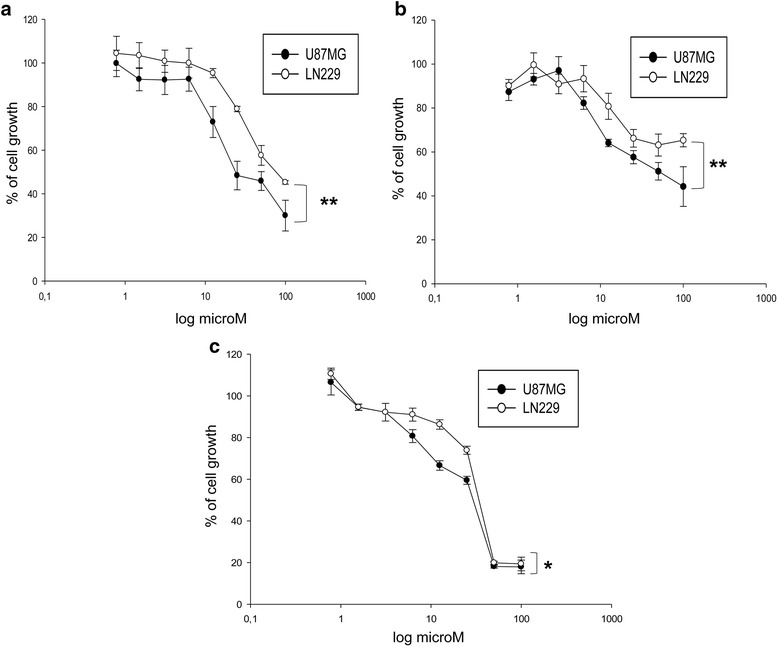
Effects of RF-Id (**a**), RF-Idmet(**b**) and embelin(**c**) on cell growth inhibition. Human GBM cells U87MG and LN229 were seeded in serum-containing media in 96-well plates at the density of 2 × 10^3^ cells/well. After 24 h incubation at 37 °C, cells were treated with increasing concentrations of RF-Id (**a**), RF-Idmet (**b**) and embelin (**c**) (0,8–100 μM) for 72 h. Cell viability was assessed by MTT assay as described in Material and methods. ** *p* ≤ 0.01

In order to investigate the antitumor activity of the new benzoquinone derivatives, we evaluated the effects of **RF-Id**, **RF-Idmet** and embelin on cell growth of two human GBM cell lines (U87MG and LN229) after 24 h, 48 h and 72 h of treatment. Cell growth inhibition was evaluated by cell viability assay as described in “Materials and methods” and resulted time- and dose-dependent for all compounds. In details, after 72 h **RF-Id** and RF-Idmet induced 50 % (IC:50) of growth inhibition at a concentration of 23.6 and 47.5 μM in the U87MG and 77 and 100 μM in LN229, respectively while IC:50 of embelin was 30 μM in U87MG and 33 μM in LN229 (Fig. 1).

On the other hand, NDGA, a natural 5-LOX inhibitor and its methylated derivative terameprocol inhibited 50 % of cell growth at a concentration of 87 and >50 μM in U87MG and >50 μM in LN229, respectively, as reported in Table 1. In conclusion, **RF-Id** was more potent than its methylated derivative and embelin in inducing growth inhibition on U87MG cells.

**Table 1:**
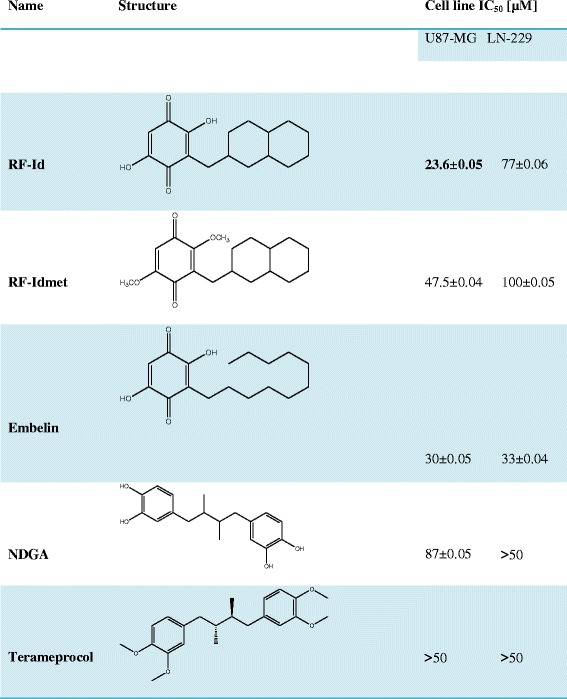
IC:50 values of the different compounds in U87MG and LN229 cells after 72 h of treatment

### Effects of RF-Id on cell cycle modulation

In order to study the molecular mechanisms of the antiproliferative activity of **RF-Id,** we performed cell cycle analysis by flow cytometry. On the basis of the IC:50 values, U87MG cells resulted more sensitive to the treatment with **RF-Id** and were selected for subsequent experiments. Cells were treated for 72 h with **RF-Id** and, subsequently, were stained with propidium iodide for 1 h and analyzed by flow cytometry. For each sample, the percentage of cells in each phase of the cell cycle (G0/G1-S-G2M) was calculated.

**Fig. 2:**
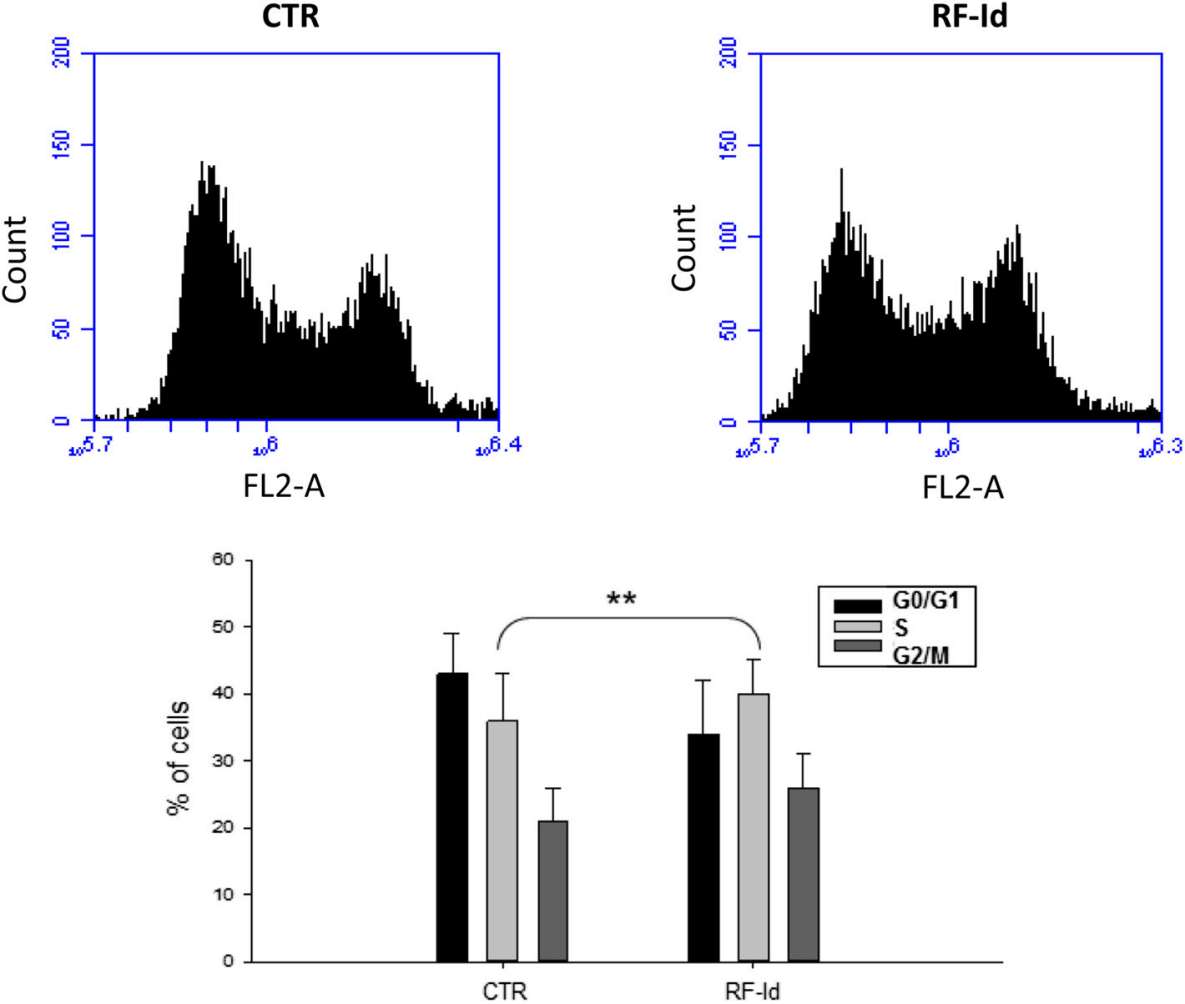
Effects of RF-Id on cell cycle. U87-MG cells were treated for 72 h with IC:50 of **RF-Id** and subsequently were stained with propidium iodide for 1 h and analyzed by flow cytometry. For each sample, the percentage of cells in each phase of the cell cycle (G0 / G1-S-G2M) was calculated. The experiments were repeated three times giving always similar results. Bars, SDs. ** *p* ≤ 0.01

Cell cycle analysis showed that treatment with **RF-Id** induced an increase of S and G2/M cells and a decrease of G0/G1 (Fig. 2). In details, as reported in the histogram, the percentage of cells in S phase and G2/M was equal to 35 and 20 %, respectively, in the control while it increased (about 45 and 30 %, respectively) with the treatment; on the other hand, the percentage of cells in G0/G1 decreased from 45 to 35 % after treatment with **RF-Id**.

### Effects of RF-Id on apoptosis

**Fig. 3:**
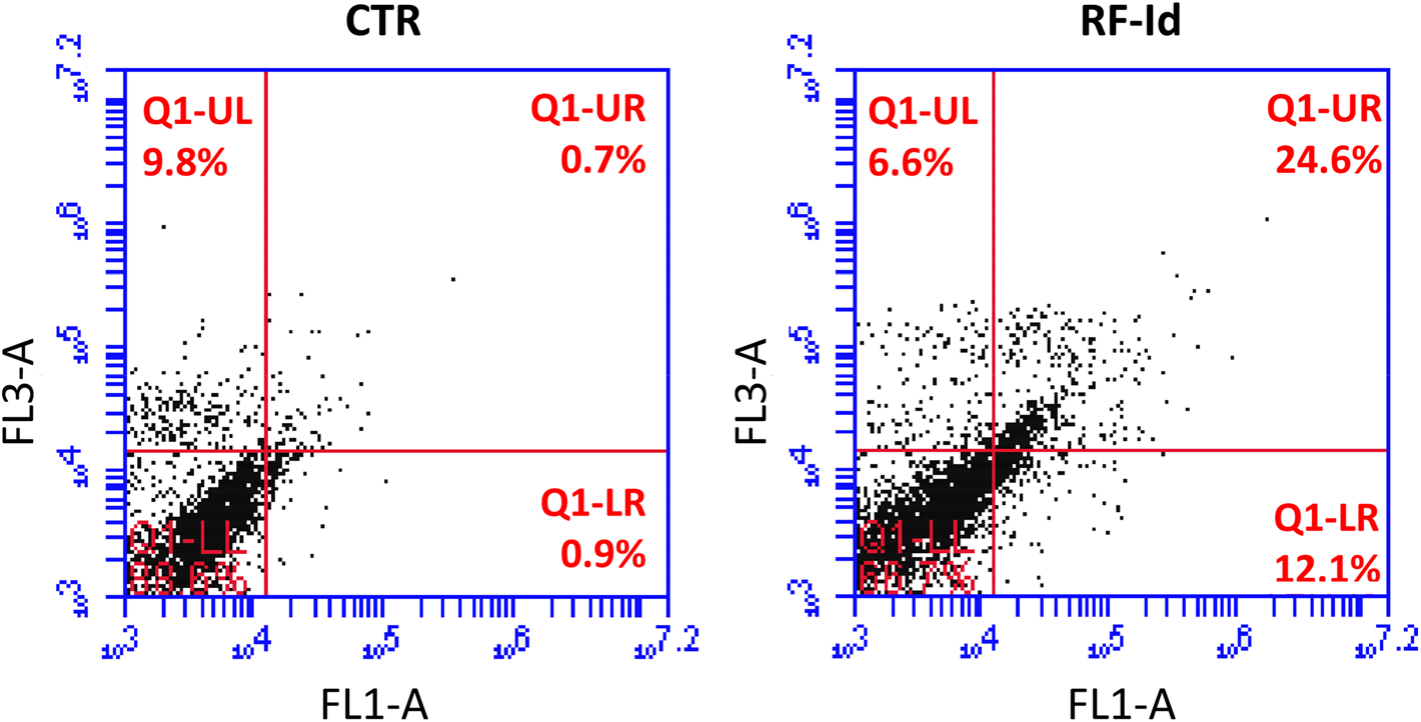
Effects of RF-Id on apoptosis. U87 MG cells were not treated or treated with **RF-Id** IC:50 for 72 h. Apoptosis was evaluated by FACS analysis, after double cell labeling with propidium iodide (PI) and Annexin V-FITC. The lower left quadrants of each panel show the viable cells, which exclude PI and are negative for AnnexinV-FITC binding. The upper left quadrants contain the non-viable, necrotic cells, negative for Annexin V-FITC binding and positive for PI uptake. The lower right quadrants represent cells in early apoptosis that are Annexin V-FITC positive and PI negative. The upper right quadrants represent the cells in late apoptosis, positive for both Annexin V-FITC binding and for PI uptake. The experiments were performed at least three times and the results were always similar

Subsequently, we evaluated the effects of **RF-Id** on apoptosis by flow cytometry. After 72 h of treatment, U87MG cells were stained with Annexin V-FITC and propidium iodide and analyzed by flow cytometry. As shown in Fig. 3, **RF-Id** induced about 30 % of apoptosis compared to control cells (1,6 %); in details, 24 % of the cells were in late apoptosis and 12 % in early apoptosis. No significant effects were recorded on necrosis. In conclusion, **RF-Id** showed a significant pro-apoptotic effect.

**Fig. 4:**
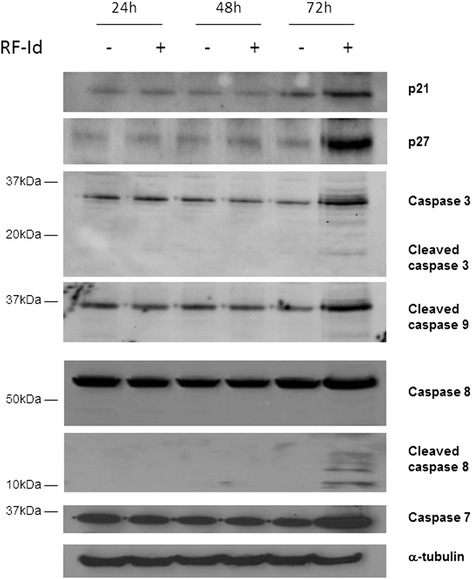
Effects of RF-Id on signal transduction pathways. U87MG cells were treated for 72 h with IC:50 of **RF-Id**. Cell lysates were incubated with anti-human caspases 8, 3, 7, 9, p21 and p27 antibodies and analysed by Western Blotting; the housekeeping protein α-tubulin was used as loading control. The experiments were repeated three times giving always similar results. Quantitation of the bands for each proteins are reported in Additional file 1: Figure S3

In order to confirm the apoptotic effect induced by **RF-Id** on U87MG cells, we examined its effects on caspase cascade by Western blotting technique (Fig. 4). In details, the cells treated for 24, 48, 72 h as described in Materials and Methods, were lysed and proteic lysates were analyzed by Western blotting. After 24 and 48 h of treatment no significant effects on the expression of caspases was observed while after 72 h **RF-Id** induced an increase of procaspases 3, 7, 8 and 9 and a significant increase of the cleaved fragments, indicative of caspase activation (Fig. 4). In addition, treatment with **RF-Id** did not alter the levels of p21 and p27 after 24 and 48 h while they significantly increased after 72 h (Fig. 4). This effect on p21 and p27 expression was likely due to a decrease of protein ubiquitination and consequently to p21 and p27 accumulation. In fact, we showed (Additional file 1: Figure S1) that after 72 h, Rf-Id treatment decreased ubiquitinated form of p21. In conclusion, **RF-Id** was able to induce apoptosis by a caspase-dependent mechanism.

### Effects of RF-Id on mitochondrial membrane potential and autophagy

**Fig. 5:**
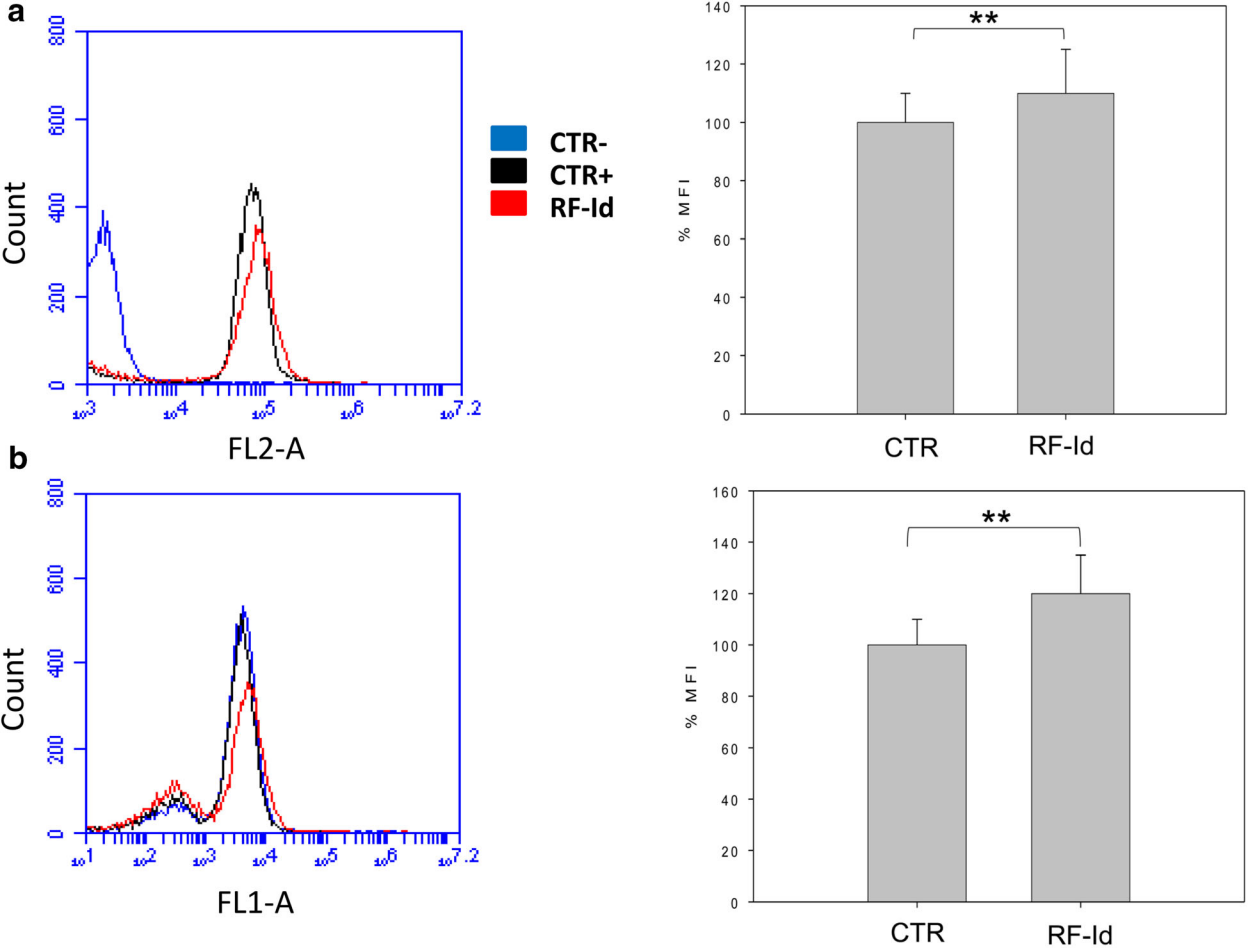
Effects of RF-Id on mitochondrial membrane potential and autophagy. Flow cytometric analysis of mitochondrial membrane potential by using Mitotracker red, dye which localized in mitochondria and changed in according to mitochondrial membrane potential variations due to oxidative stress. U87 MG cells were treated with **RF-Id** for 72 h. (*Right panels*) The %MFIs of control were calculated, as described in ‘Materials and Methods’ and represented as columns. The experiments were performed at least three times and the results were always similar. Bars, SDs.** *p* ≤ 0.01 (**a**) Flow cytometric analysis of autophagosome formation (MDC incorporation) in U87 MG cells treated with RF-Id for 72 h. (*Right panels*) The %MFIs of control were calculated, as described in ‘Materials and Methods’ and represented as columns. The experiments were performed at least three times and the results were always similar. Bars, SDs. ** p ≤ 0.01

We evaluated the effects of **RF-Id** on mitochondrial membrane potential by flow cytometry. Cells were treated for 72 h at concentrations equal to IC:50 and stained with Mitotracker dye which localized in mitochondria and changed in according to mitochondrial membrane potential variations due to oxidative stress. **RF-Id** did not induce significant effects (110 % MFI) on the membrane potential of U87MG cells compared to control (100 % MFI) (Fig. 5a). In addition, we evaluated the effects of **RF-Id** on autophagy.

In details, cells were treated for 72 h and subsequently were stained with MDC, specific marker for autophagosomes and analyzed by flow cytometry. Analysis of the data showed that treatment with **RF-Id** induced a slight increase of autophagy in U87MG (120 % MFI) compared to control (100 % MFI) (Fig. 5b).

### Identification of RF-Id regulated genes in apoptosis

**Fig. 6:**
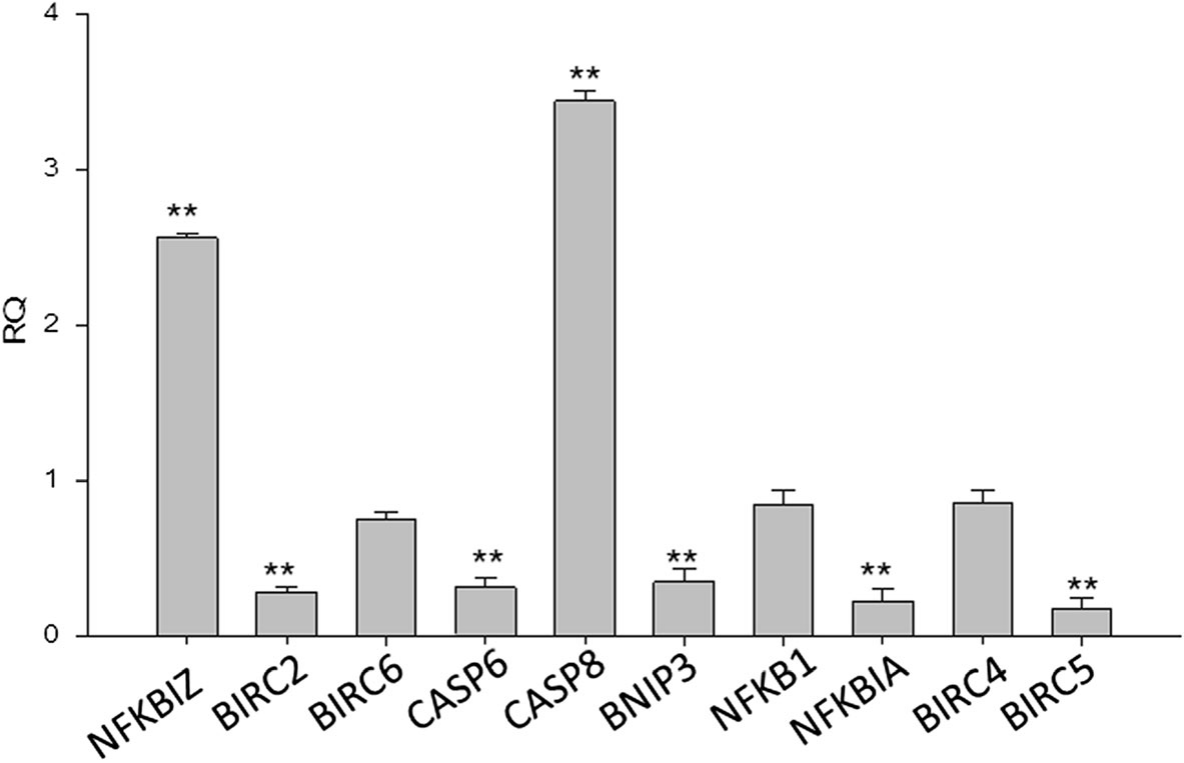
RF-Id -regulated genes in U87MG cells. RNA was prepared from unexposed U87MG cells and exposed to **RF-Id** for 72 h. Data were normalized using GAPDH, 18S and ACTB genes as internal control. Three biological replicates were performed per group. Relative expression of the transcripts was measured by using ViiA7™Real-Time PCR software. Bars, SDs.** *p* ≤ 0.01

On the basis of the data demonstrating that **RF-Id** induced caspase-dependent cell death in U87MG cells, we assessed more globally the mechanisms involved in **RF-Id**-induced apoptosis using microarray analysis. Therefore, RNA was prepared from either untreated U87MG cells or cells exposed to **RF-Id** for 72 h. Three biological replicates were performed per group. Data were normalized using the glyceraldehyde-3-phosphate dehydrogenase (GAPDH), ACTB and 18S genes as internal control. After 72 h of treatment, differential gene expression analysis revealed 10 significantly modulated transcripts (8 decreased and 2 increased) in **RF-Id**-treated cells compared to controls. In particular, *BIRC2*, *BIRC4*, *BIRC5* and *BIRC6*, *NFκB1*, *NFκBIA*, *CASP6* and *BNIP3* were downregulated. At this time, *CASP8* and *NFκBIZ* were the only upregulated genes detected in this analysis (Fig. 6). Other genes did not show highly significant changes as reported in Additional file 1: Table S1. Intriguingly, most of the upregulated genes were associated to extrinsic pathway (*CASP8*) while downregulated genes belonged to *IAP* family and *NFκB* pathway.

### RF-Id inhibits XIAP-cIAP2 interaction

**Fig. 7:**
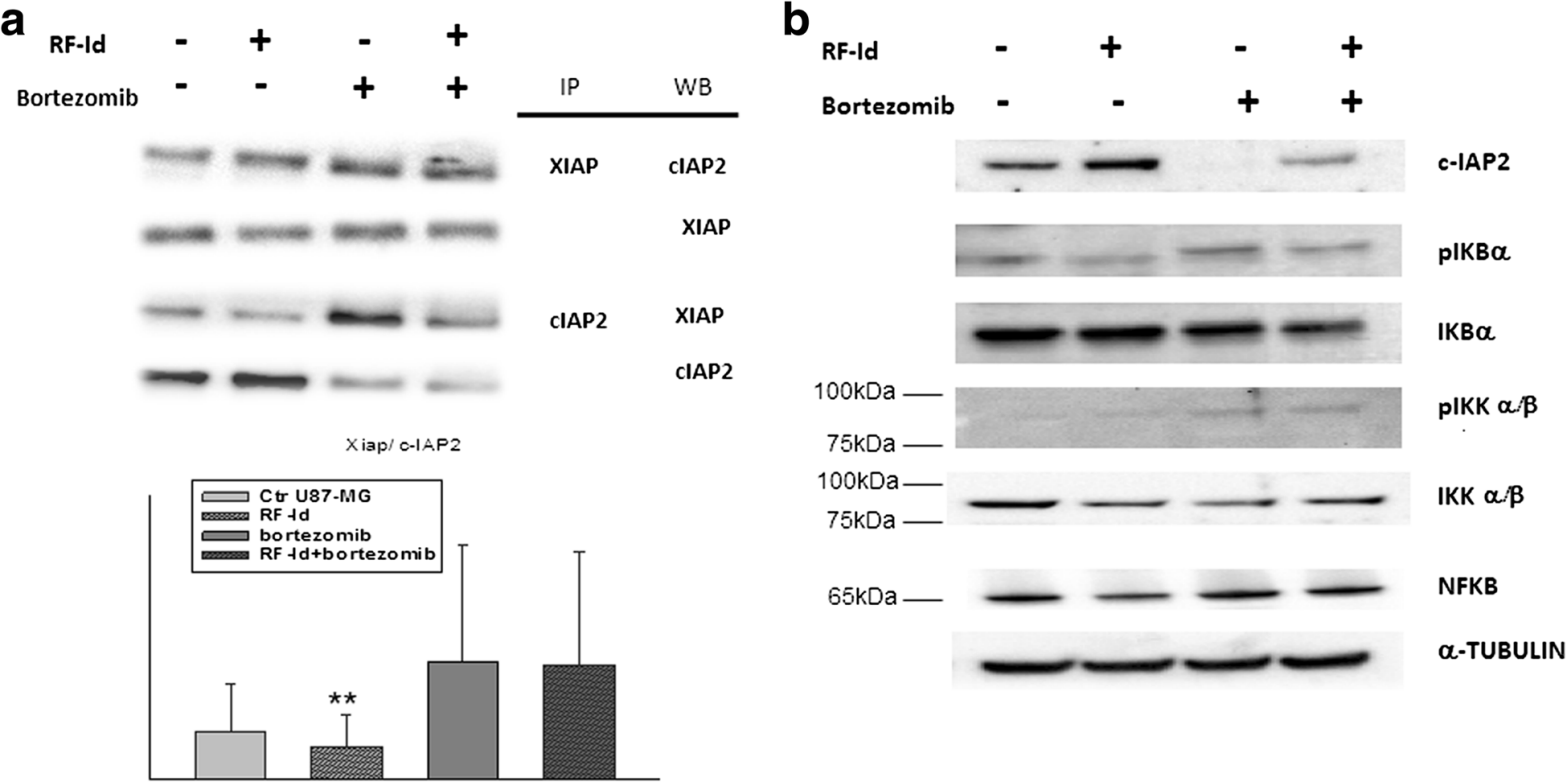
RF-Id inhibits XIAP-cIAP2 interaction and NFκB activation. Total protein extracts were subjected to immunoprecipitation with 2 μg of anti-XIAP or anti-cIAP2 for 24 h at 4 °C. Immune complexes were collected with 50 μl of protein A-agarose for 16 h at 4 °C. The protein A-agarose/immune complex was washed twice with cold PBS, resuspended in 20 μl of SDS-loading buffer, heated to 95 °C for 5 min and used for Western blotting analysis using anti-XIAP or anti-CIAP2 . Representation of the c-IAP2 and XIAP complexes was expressed as the mean of the ratio between the relative intensities of the bands associated with the c-IAP2/XIAP complexes versus the bands associated with total C-IAP2 and XIAP, respectively. The intensities of the bands were expressed as arbitrary units when compared to that of the untreated cells. Error bars showed standard deviation from the mean in at least three independent experiments. Bars, SDs.** *p* ≤ 0.01 (**a**). U87MG cells were treated for 72 h with IC:50 of RF-Id or 500nM Bortezomib for 24 h or a combination of RF-Id and Bortezomib. Cell lysates were incubated with anti-human c-IAP2, pIKKα/β, IKKα/β, pIKBα, IKBα and NFκB antibodies and analysed by Western Blotting; the housekeeping protein α-tubulin was used as loading control. The experiments were repeated three times giving always similar results. Quantitation of the bands for each proteins are reported in Additional file 1: Figure S4 (**b**)

Since **RF-Id** dowregulated genes belonged to IAP family and NFκB pathway and embelin inhibits the X-linked inhibitor of apoptosis protein (XIAP) by binding to its Baculovirus Inhibitor of apoptosis protein Repeat (BIR) domain, we investigated the action mechanism of the compound. Recent evidence showed that XIAP stabilizes c-IAP2 at the protein level by inhibiting its auto-ubiquitination and the increased expression of c-IAP2 enhances the phosphorylation of IκBα that favours its subsequent proteasomal degradation leading to activation of the canonical NFκB pathway. In order to investigate if **RF-Id** disrupts XIAP-cIAP2 complex by inhibiting NFκB pathway, we evaluated the effects of **RF-Id** on either XIAP, cIAP2 and XIAP –cIAP2 complex expression and on IKKα/β, IKBα and NFκB activation and expression. After 72 h **RF-Id** induced an increase of c-IAP2, a decrease of XIAP and significantly reduced the complex (Fig. 7a). Probably, **RF-Id** reduced XIAP by increasing its degradation, since bortezomib blocked **RF-Id** mediated-XIAP degradation and increased its expression. Moreover, **RF-Id** reduced IKKα/β and IkBα phosphorilation by increasing IKBα expression thus inhibiting NFκB (Fig. 7b). On the other hand, bortezomib antagonized the effects of **RF-Id** by increasing XIAP–cIAP-2 complex and leading to IKKα/β-mediated IkBα phosphorylation and degradation (Fig. 7b). Moreover, Rf-Id mediated both apoptosis and caspase activation through degradation of XIAP, that acts as apoptosis inhibitor by binding caspases 9, 3 and 7. Bortezomib antagonized Rf-Id-mediated caspase activation as it blocked XIAP degradation making XIAP able to inhibit both caspases activity and apoptosis identified as “DNA ladder” by agarose gel electrophoresis (Additional file 1: Figure S2).

In conclusion, **RF-Id** inhibited cIAP2-XIAP complex formation and IkBα degradation by leading to NFκB inhibition and caspase activation.

## Discussion

Several benzoquinones have been found effective in treating some forms of cancer [17]; in details, it has been shown that these compounds act on cells by regulating numerous mechanisms, such as apoptosis, cell cycle [30], interacting with human-telomeric G-quadruplex DNA [31] or producing reactive oxygen species (ROS) [32]. Our group has been interested for a long time in synthesis and biological evaluation of anti-inflammatory and anti-cancer agents including quinone-based compounds [20–23]. Within the context of our investigations on quinone derivatives and on their possible therapeutic use, we recently studied the natural compound embelin and the synthetic derivative **RF-Id [** 15, 19]. Previous research has demonstrated that embelin, a small inhibitor binding the Baculovirus Inhibitor of apoptosis protein Repeat (BIR) domain of the X-linked inhibitor of apoptosis protein (XIAP), suppressed proliferation of human glioma cells and induced apoptosis by inhibiting NF-κB signaling pathway. Our previous work revealed embelin as potent dual inhibitor of 5-LOX and microsomal prostaglandin E_2_ synthase (mPGES)-1 with IC_50_ values of 60 and 200 nM in cell-free assays, respectively. Moreover **RF-Id**, demonstrated an improved efficiency against 5-LOX in human neutrophils (IC_50_ = 580 nM) and in vivo anti-inflammatory activity.

The high potency on 5-LOX and the promising in vivo efficiency of synthesized compound prompted us to investigate the **RF-Id** antiproliferative effects in GBM tumor cell lines.

We showed that **RF-Id** had two major time-dependent in vitro consequences on U87-MG cells. This compound modulated cell cycle arrest and drove cells to programmed cell death by inhibiting cIAP2-XIAP complex formation and IkBα degradation and leading to NFκB pathway inactivation.

First, we found that **RF-Id** was more potent than its methylated derivative and embelin in inducing growth inhibition on GBM cells, in particular on U87MG cells.

In order to study the molecular mechanisms of the antiproliferative activity of **RF-Id,** we performed cell cycle and apoptosis analysis by flow cytometry. In details, after 72 h **RF-Id** induced about 30 % apoptosis and cell death was mediated by caspases and occurred through a ROS-independent mechanism. In fact, the compound induced a significant cleavage of the initiator caspases 8, 9, and of effector caspases 3 and 7 but did not induce significant alterations of the mitochondrial membrane potential associated to increased ROS production in mitochondria. Moreover, the compound induced an increase in the percentage of cells in G2 and S phase that occurred in parallel to an increase of the expression of p21 and p27. The increase of p21 and p27 could be due to a decrease of protein ubiquitination and hence, a reduced degradation rather than to an increase of gene transcription. In fact, XIAP or c-IAP2 may act as E3-ubiquitin ligases promoting the degradation of CDK inhibitors, p21 and p27 [33]. We recorded a late increase of p21 and p27 expression after treatment with Rf-Id that could be due to a decrease of protein ubiquitination and consequently to p21 and p27 accumulation. This effect on p21 and p27 expression can be likely ascribed to RF-Id that induces XIAP degradation and c-IAP2 inhibition, blocks their ubiquitin ligases activity and reduces p21 and p27 degradation by proteosome. As shown in Additional file 1, we found a decrease of ubiquitinated form of p21 after 72 h from the beginning of Rf-Id treatment.

In addition, **RF-Id** induced only a slight 20%increase of autophagy that may be due to a blockage of the autophagic flow that causes an accumulation of MDC, marker of autophagosomes. The action mechanism of **RF-Id** was confirmed by performing apoptosis array. Data analysis, in fact, showed a significant upregulation of *CASP8* and a downregulation of *IAP* family proteins (*BIRC2, 4,5,6* and *7*) and *NFκB* in cells treated with **RF-Id** for 72 h. Probably, **RF-Id** acts by inhibiting *IAP* family genes (*XIAP*, *c-IAP1*, *c-IAP2*) and NFκB pathway and inducing apoptosis through both intrinsic and extrinsic pathways thus, resulting in the amplification of the apoptotic signal.

Recent evidence showed that XIAP stabilizes c-IAP2 at the protein level by inhibiting its auto-ubiquitination and the increased expression of c-IAP2 enhances the phosphorylation of IκBα that favours its subsequent proteasomal degradation leading to activation of the canonical NFκB pathway [34, 35]. In fact, we hypothesized that RF-1d by inhibiting XIAP could decrease c-IAP2, but we found that c-IAP2 was indeed increased. Based on these results, we suggest that RF-Id could disrupt XIAP-cIAP2 binding by inducing XIAP ubiquitination and proteosome-mediated degradation as bortezomib blocked RF-Id-mediated XIAP degradation and increased its expression. On this light, RF-Id could bind and stabilize c-IAP2 at the protein level by displacing XIAP but inhibits its activity as caspase inhibitor by blocking BIR3 domain of c-IAP2, as a consequence we found that RF-Id activated caspases 3, 7, 8 and 9. Binding of the XIAP RING domain to c-IAP2 is a pre-requisite for stabilization of c-IAP2, even though the RING activity may be defective, suggesting that occupation of BIR domains 2 and 3 on c-IAP2 by XIAP through its RING finger guarantees the ensuing increase in c-IAP2 stability. Protein interactions occur through binding of BIR2 and BIR3 domains of c-IAP2 with the RING finger of XIAP. RF-Id probably binds BIR3 domain of c-IAP2 and stabilizes it by blocking its degradation but inhibits its caspase inhibitor activity so that the BIR-bound caspases are released and reactivated; in the same time, Rf-Id displaces XIAP that undergoes to auto-ubiquitination and is degraded via proteosome. Moreover, Rf-Id acts inhibiting c-IAP1 while c-IAP2 undergoes to a posttranscriptional regulation; it increases because its degradation is reduced. In fact, it is reported that cells from c-IAP1(-/-) mice express markedly elevated levels of c-IAP2 protein in the absence of increased c-IAP2 mRNA. Transient transfection studies with wild-type and E3-defective c-IAP1 revealed that c-IAP2 is a direct target for c-IAP1-mediated ubiquitination and subsequent degradation [36].

**Fig. 8:**
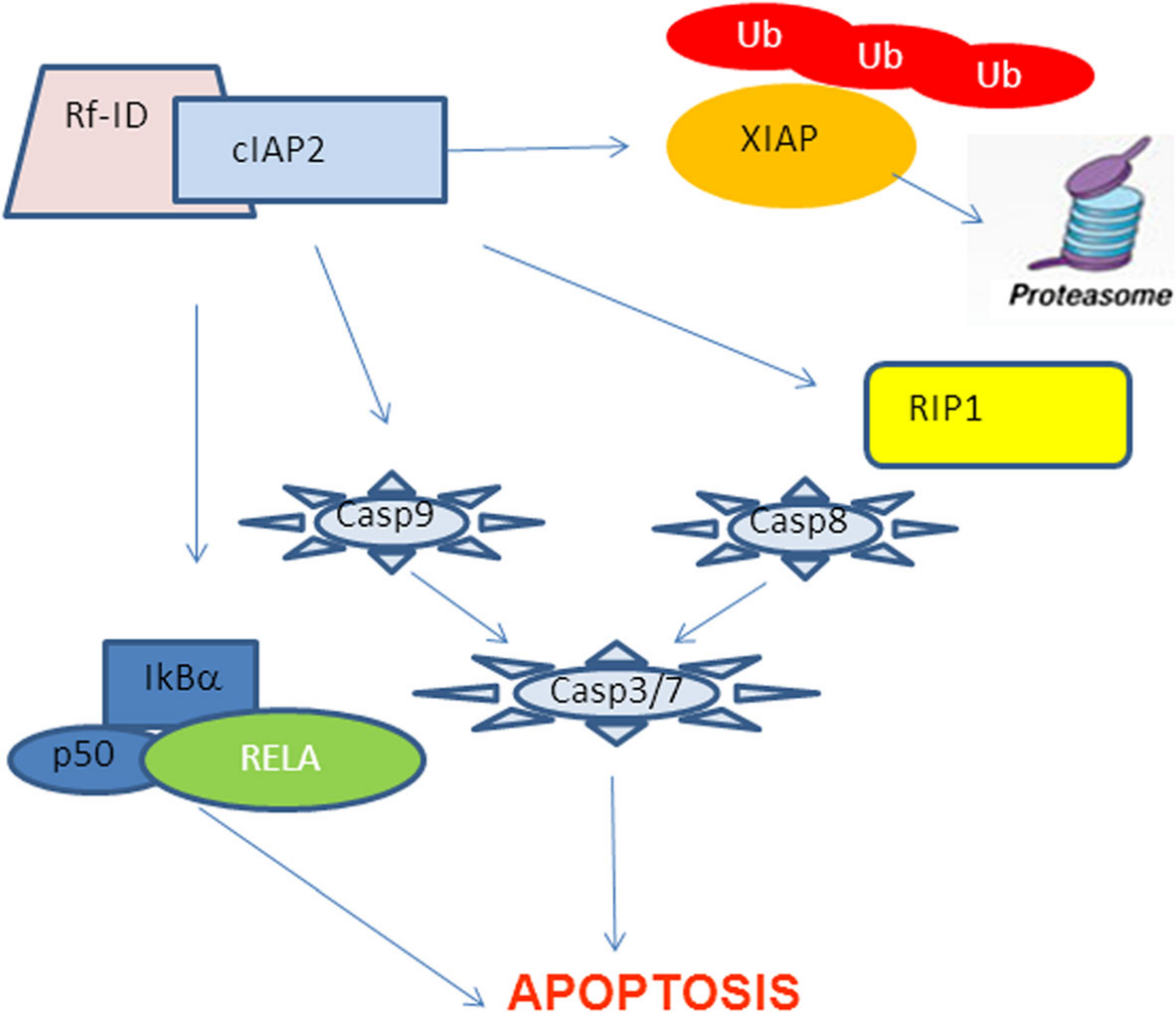
RF-Id inhibits cIAP2 and XIAP activity and NFκB pathway. **RF-Id** disrupts XIAP-cIAP2 binding by inducing XIAP ubiquitination and proteosome-mediated degradation. Moreover, **RF-Id** stabilizes c-IAP2 at the protein level but inhibits its activity as caspases inhibitor by blocking BIR3 domain of cIAP2, thus activating caspases 3,7, 8 and 9. Moreover, blockade of cIAP2-XIAP complex formation increases IkBα expression leading to NFκB inactivation and apoptosis induction

Blockade of cIAP2-XIAP complex formation increased IkBα expression leading to NFκB inhibition and apoptosis induction by reducing survivin and FLIP, a caspase 8 inhibitor as confirmed by microarray analysis (Fig. 8).

On the other hand, bortezomib antagonized the effects of **RF-Id** by increasing XIAP–cIAP2 complex leading to IKKα/β-mediated IKBα phosphorylation and NFκB activation as it blocked **RF-Id-**mediated XIAP degradation [37]. As reported in the literature [38], Bortezomib downregulated cIAP2 expression, probably by a NFκB-independent mechanism. Moreover, Rf-Id mediated both apoptosis and caspase activation through degradation of XIAP, that acts as apoptosis inhibitor by binding caspases 9, 3 and 7. Bortezomib antagonized Rf-Id-mediated caspase activation as it blocked XIAP degradation making XIAP able to inhibit caspase-mediated apoptosis.

## Conclusions

**RF-Id** induced apoptosis associated with modulation of cell cycle on U87MG by inducing XIAP degradation and inhibiting NFκB pathway. In conclusion, **RF-Id** could represent a new therapeutic strategy in GBM, due to its highly lipophilic structure, able to target multiple intracellular components.

## Additional file﻿﻿﻿

Additional file 1:
**Figure S1**. Rf-Id induced p21 accumulation through a decrease of its ubiquitination. U87MG cells were treated for 72h with IC:50 of RF-Id. Total protein extracts were subjected to immunoprecipitation with 2 μg of anti-p21 for 24 h at 4 °C. Immune complexes were collected with 50 μl of protein A-agarose for 16 h at 4 °C. The protein A-agarose/immune complex was washed twice with cold PBS, resuspended in 20 μl of SDS-loading buffer, heated to 95 °C for 5 min and used for Western blotting analysis using anti-ubiquitin antibody.(Ub-p21: ubiquitinated p21). **Figure S2**. Rf-Id induced apoptosis in glioblastoma cells. U87MG were untreated (Lane 1) or treated with 72h with IC:50 of RF-Id (Lane2) or 500 nM Bortezomib for 24h (Lane 3) or a combination of RF-Id and Bortezomib (Lane 4). Chromosomal DNA was prepared using the Quick Apoptotic DNA ladder Detection kit according to the kit instructions. 10 μL of each sample was electrophoresed on a 1.5% agarose/EtBr gel (A). U87MG cells were treated for 72h with IC:50 of RF-Id or 500 nM Bortezomib for 24h or a combination of RF-Id and Bortezomib. Cell lysates were incubated with anti-human XIAP, caspase 3 and 7 antibodies and analysed by Western Blotting; the housekeeping protein α-tubulin was used as loading control. The experiments were repeated three times giving always similar results (B). **Figure S3** Effects of RF-Id on signal transduction pathway. The intensities of the bands were expressed as arbitrary units when compared to that of the untreated cells and normalized for α-tubulin. Error bars showed standard deviation from the mean in at least three independent experiments. Bars, SDs .** p≤0.01 **Figure S4** RF-Id inhibits XIAP-cIAP2 interaction and NFκB activation. The intensities of the bands were expressed as arbitrary units when compared to that of the untreated cells and normalized for α-tubulin. Error bars showed standard deviation from the mean in at least three independent experiments. Bars, SDs.** p≤0.01. (DOC 654 kb)
