# Supplementary material for: The 1,4 benzoquinone-featured 5-lipoxygenase inhibitor RF-Id induces apoptotic death through downregulation of IAPs in human glioblastoma cells
Source: J Exp Clin Cancer Res. 2016 Oct 22;35:167. doi: 10.1186/s13046-016-0440-x (PMC5075202; doi:10.1186/s13046-016-0440-x)
Supplement: Additional file 1: — Figure S1. Rf-Id induced p21 accumulation through a decrease of its ubiquitination. U87MG cells were treated for 72h with IC:50 of RF-Id. Total protein extracts were subjected to immunoprecipitation with 2 μg of anti-p21 for 24 h at 4 °C. Immune complexes were collected with 50 μl of protein A-agarose for 16 h at 4 °C. The protein A-agarose/immune complex was washed twice with cold PBS, resuspended in 20 μl of SDS-loading buffer, heated to 95 °C for 5 min and used for Western blotting analysis using anti-ubiquitin antibody.(Ub-p21: ubiquitinated p21). Figure S2. Rf-Id induced apoptosis in glioblastoma cells. U87MG were untreated (Lane 1) or treated with 72h with IC:50 of RF-Id (Lane2) or 500 nM Bortezomib for 24h (Lane 3) or a combination of RF-Id and Bortezomib (Lane 4). Chromosomal DNA was prepared using the Quick Apoptotic DNA ladder Detection kit according to the kit instructions. 10 μL of each sample was electrophoresed on a 1.5% agarose/EtBr gel (A). U87MG cells were treated for 72h with IC:50 of RF-Id or 500 nM Bortezomib for 24h or a combination of RF-Id and Bortezomib. Cell lysates were incubated with anti-human XIAP, caspase 3 and 7 antibodies and analysed by Western Blotting; the housekeeping protein α-tubulin was used as loading control. The experiments were repeated three times giving always similar results (B). Figure S3 Effects of RF-Id on signal transduction pathway. The intensities of the bands were expressed as arbitrary units when compared to that of the untreated cells and normalized for α-tubulin. Error bars showed standard deviation from the mean in at least three independent experiments. Bars, SDs .** p≤0.01 Figure S4 RF-Id inhibits XIAP-cIAP2 interaction and NFκB activation. The intensities of the bands were expressed as arbitrary units when compared to that of the untreated cells and normalized for α-tubulin. Error bars showed standard deviation from the mean in at least three independent experiments. Bars, SDs.** p≤0.01. (DOC 654 kb) [file 13046_2016_440_MOESM1_ESM.doc]

**Supplementary Table 1. RF-Id -regulated genes in U87MG cells.**

| **Genes** | **RQ** |
| --- | --- |
| BID | 0.68±0.02 |
| BIRC4 | 0.86±0.05 |
| BIRC5 | 0.17±0.02 |
| BNIP3 | 0.35±0.03 |
| NFKB1 | 0.85±0.05 |
| NFKB1A | 0.22±0.02 |
| PEA15 | 1.05±0.03 |
| PMAIP1 | 1.22±0.02 |
| REL-A | 0.63±0.04 |
| RIPK1 | 0.37±0.03 |
| RIPK2 | 0.92±0.05 |
| CASP7 | 0.48±0.2 |
| CASP8 | 3.44±0.04 |
| HIP1 | 1.43±0.05 |
| BCL2A1 | 0.74±0.03 |
| BCL10 | 3.01±0.02 |
| CASP4 | 1.63±0.02 |
| TNFRSF10B | 0.98±0.05 |
| NFKB1Z | 2.56±0.02 |
| TNFRSF1A | 0.48±0.08 |
| BIRC2 | 0.28±0.02 |
| BIRC6 | 0.75±0.04 |
| BNIP3L | 0.65±0.06 |
| CASP3 | 0.38±0.3 |
| DIABLO | 0.86±0.02 |
| HTRA2 | 0.73±0.02 |
| TBK1 | 0.51±0.3 |
| BAX | 0.42±0.2 |

**Supplementary figure 1**

**
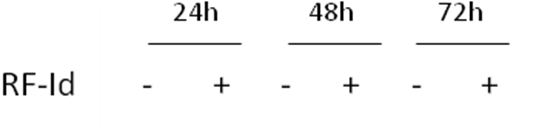
**

**
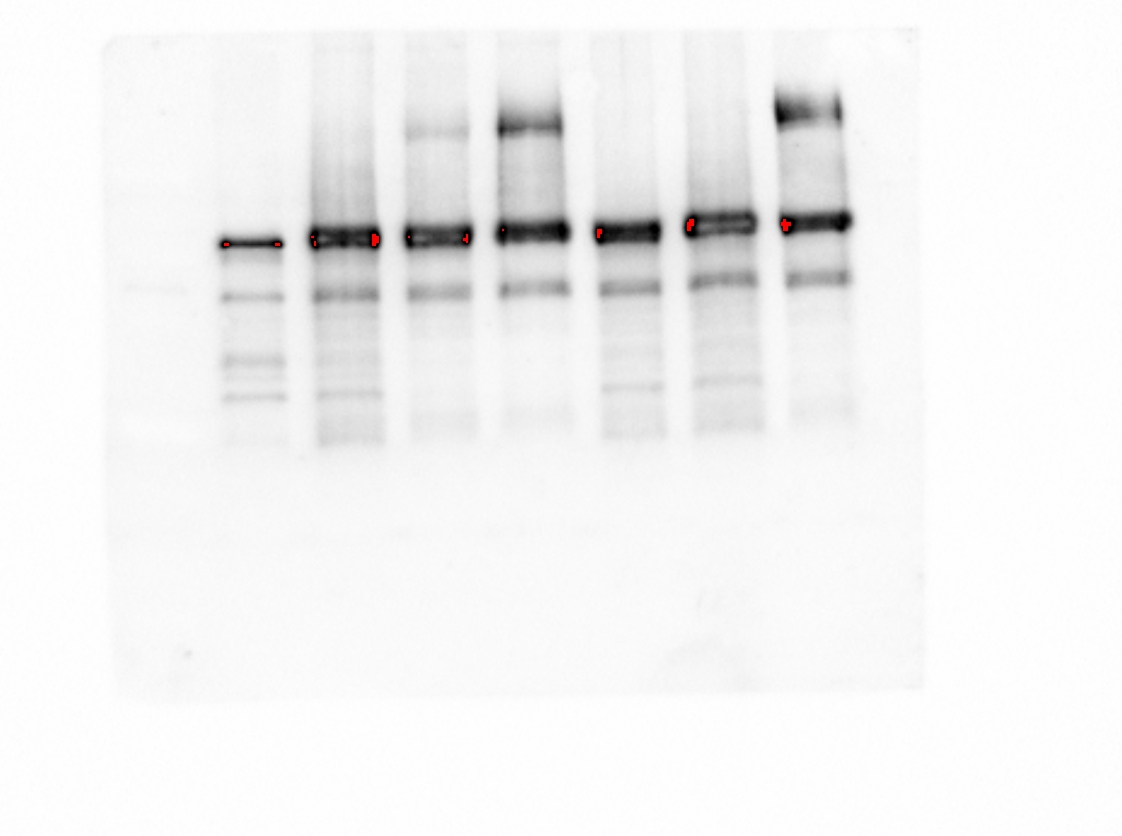
 Ub-p21**

**Rf-Id induced p21 accumulation through a decrease of its ubiquitination.**

U87MG cells were treated for 72h with IC:50 of **RF-Id**. Total protein extracts were subjected to immunoprecipitation with 2 μg of anti-p21 for 24 h at 4 °C. Immune complexes were collected with 50 μl of protein A-agarose for 16 h at 4 °C. The protein A-agarose/immune complex was washed twice with cold PBS, resuspended in 20 μl of SDS-loading buffer, heated to 95 °C for 5 min and used for Western blotting analysis using anti-ubiquitin antibody.(Ub-p21: ubiquitinated p21)

**Supplementary figure 2**

**
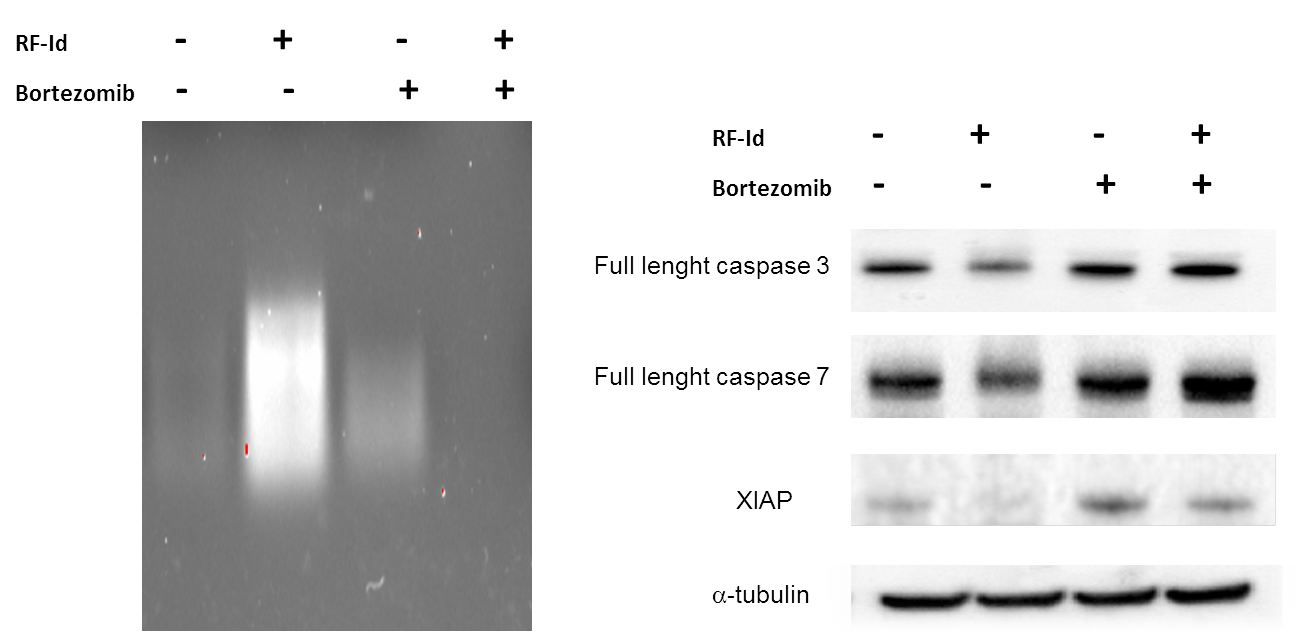
**

**Rf-Id induced apoptosis in glioblastoma cells.**

U87MG were untreated (Lane 1) or treated with 72h with IC:50 of RF-Id (Lane2) or 500nM Bortezomib for 24h (Lane 3) or a combination of RF-Id and Bortezomib (Lane 4). Chromosomal DNA was prepared using the Quick Apoptotic DNA ladder Detection kit according to the kit instructions. 10μL of each sample was electrophoresed on a 1.5% agarose/EtBr gel (A). U87MG cells were treated for 72h with IC:50 of RF-Id or 500nM Bortezomib for 24h or a combination of RF-Id and Bortezomib. Cell lysates were incubated with anti-human XIAP, caspase 3 and 7antibodies and analysed by Western Blotting; the housekeeping protein α−tubulin was used as loading control. The experiments were repeated three times giving always similar results. (B)

**Supplementary figure 3**

**
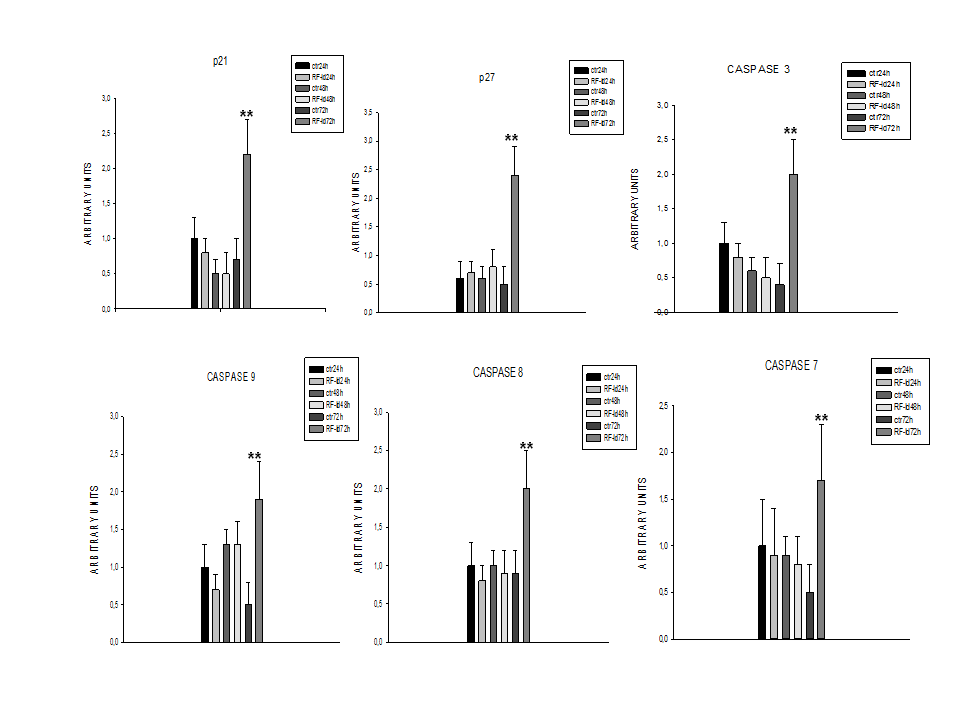
**

**Effects of RF-Id on signal transduction pathway**

The intensities of the bands were expressed as arbitrary units when compared to that of the untreated cells and normalized for α−tubulin. Error bars showed standard deviation from the mean in at least three independent experiments. Bars, SDs .** p≤0.01

**Supplementary figure 4**

**
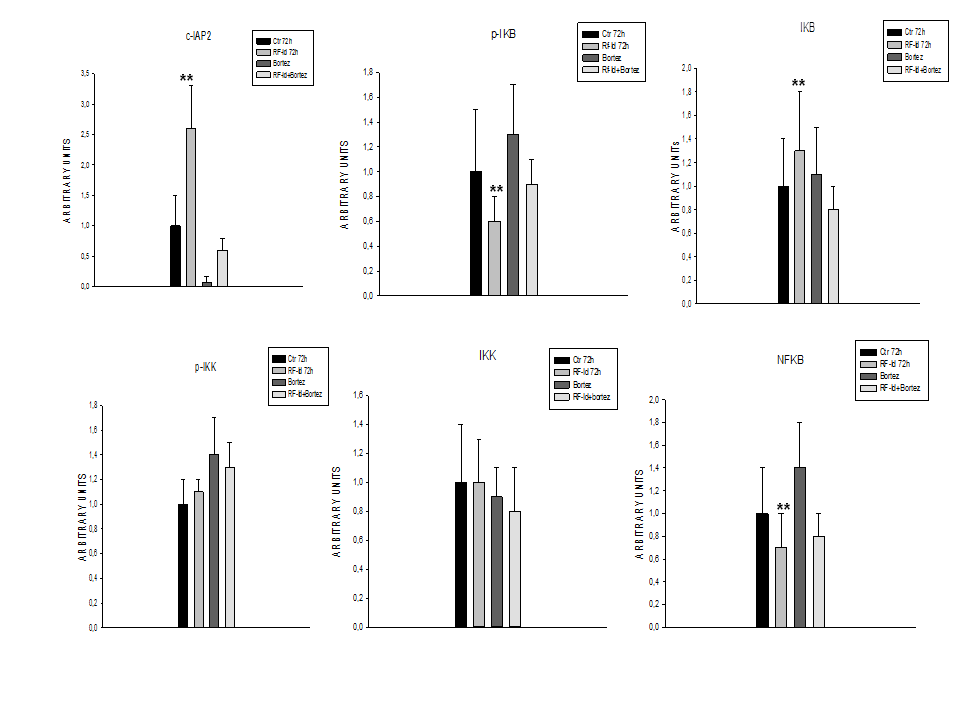
**

**RF-Id inhibits XIAP-cIAP2 interaction and NFκB activation.**

The intensities of the bands were expressed as arbitrary units when compared to that of the untreated cells and normalized for α−tubulin. Error bars showed standard deviation from the mean in at least three independent experiments. Bars, SDs .** p≤0.01
